# Supplementary material for: Direct Readout of Excited-State Lifetimes in Chlorin Chromophores under Electronic Strong Coupling
Source: J Am Chem Soc. 2026 Feb 27;148(9):9737–53. doi: 10.1021/jacs.5c21433 (PMC12983312; doi:10.1021/jacs.5c21433)
Supplement: Supplementary file 2 [file ja5c21433_si_002.pdf]

- SUPPORTING INFORMATION -

Direct readout of excited-state lifetimes in chlorin chromophores under electronic strong coupling

Alexander M. McKillop[1], Liying Chen[1], Ashley P. Fidler[1,†], and Marissa L. Weichman[1,*]

[1]Department of Chemistry, Princeton University, Princeton, New Jersey, 08544, United States

[†]Current Address: Chemistry Division, Naval Research Laboratory, Washington, DC 20375, United States

*weichman@princeton.edu

S1. Spectral characterization of ultrafast pulses

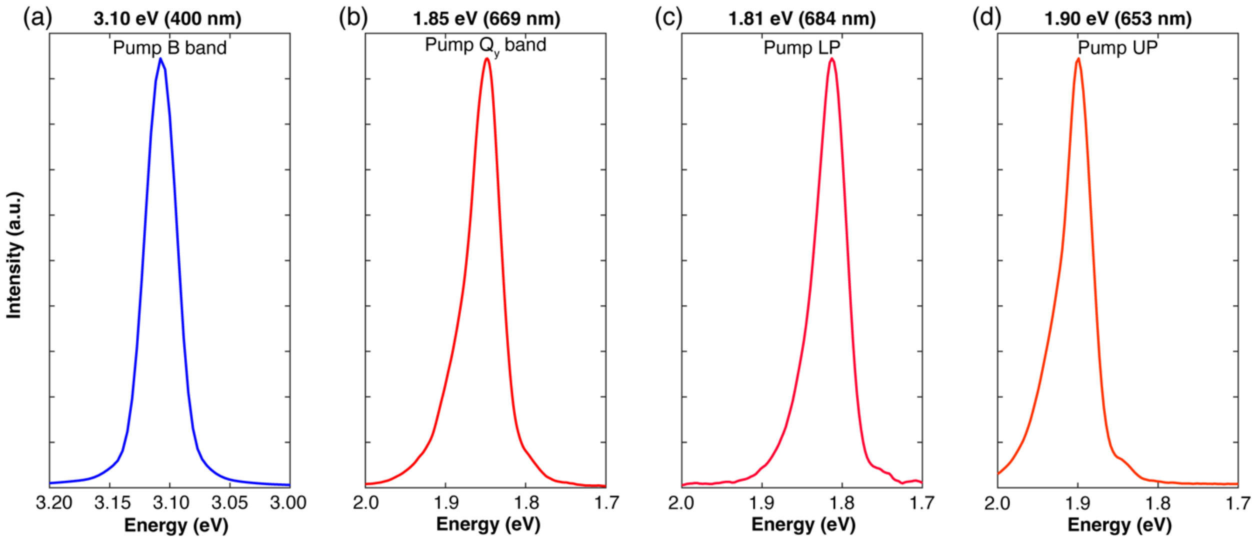

Figure S1. Spectra of pump pulses used in ultrafast pump-probe measurements. (a) Spectrum of 3.10 eV light used to pump the B band of Ce6T. (b) Spectrum of 1.85 eV light used to pump the Q_y band of Ce6T. (c) Spectrum of 1.81 eV light used to pump the lower polariton of Ce6T in Cavity 1 and in extracavity control experiments. (d) Spectrum of 1.90 eV light used to pump the upper polariton of Ce6T in Cavity 1 and in extracavity control experiments.

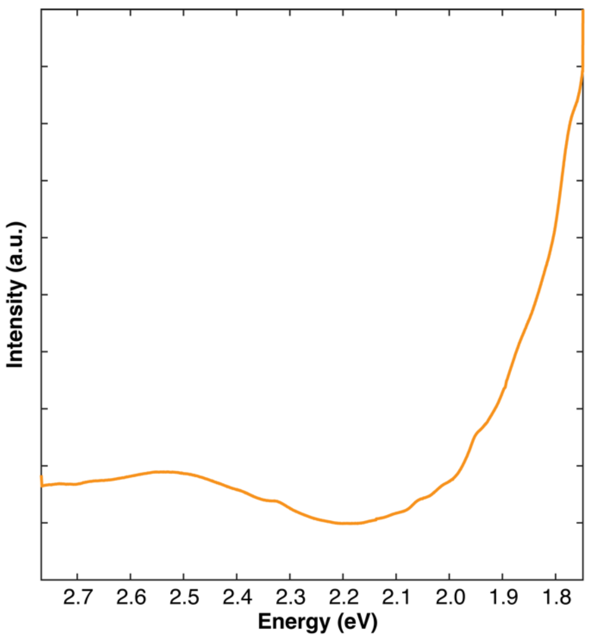

Figure S2. Spectrum of white light continuum probe pulses used in ultrafast pump-probe measurements.

S2. Characterization of the TCSPC instrument response function

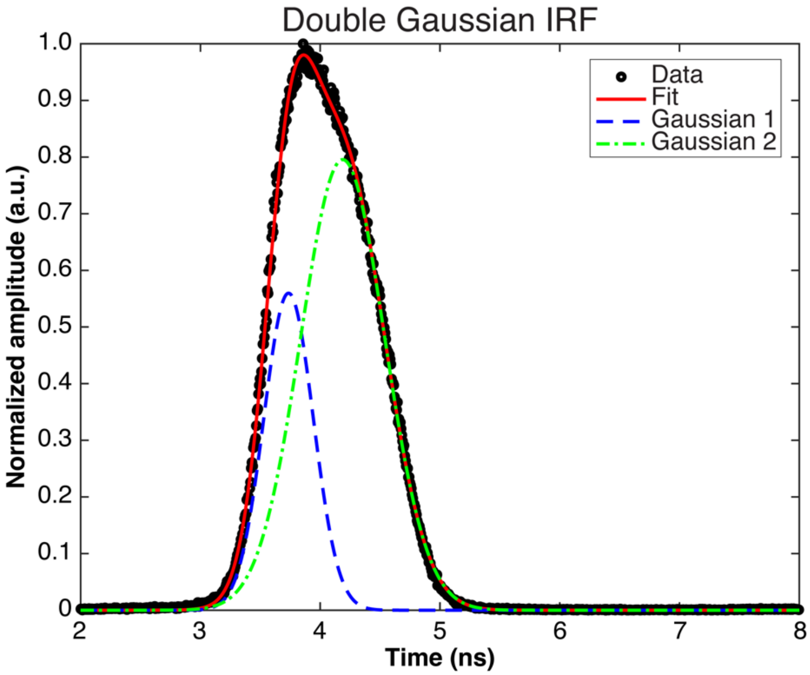

Figure S3. Ultrafast emission data from time-correlated single photon counting (TCSPC) scattering measurements of a 1.75mg/mL solution of 50 nm silica nanospheres in ethanol (nanoComposix). Data are collected using a photoluminescence spectrometer (FLS980, Edinburgh Instruments) fitted with a picosecond pulsed diode laser (EPL-405, Edinburgh Instruments) which excites the sample at 3.09 eV (401 nm) with a pulse width of 58.1 ps. We probe the scattered 3.09 eV light as a metric for the TCSPC instrument response function (IRF). The experimental data (black dots) is fit with a sum of two Gaussian lineshapes (red line) using the lsqcurvefit function in MATLAB. Gaussian 1 (blue dotted line) features a temporal width of 0.479 ns FWHM, while Gaussian 2 (green dotted line) is 0.810 ns FWHM in width. These two components feature relative amplitudes of 0.733:1, and Gaussian 2 is delayed from Gaussian 1 by 0.485 ns.

S3. Photodegradation of Ce6T films in the absence of rastering

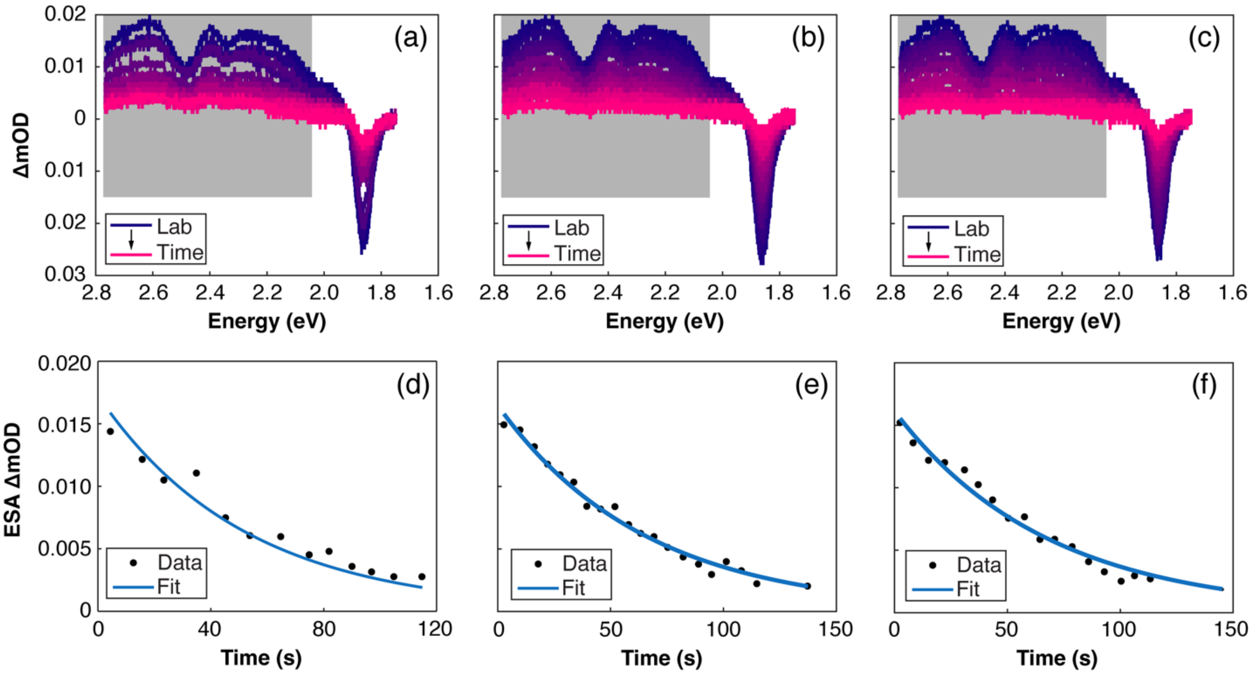

Figure S4. Decay of transient features in extracavity Ce6T/PS films under constant illumination and with no rastering, performed at three separate spatial locations. (a-c) Transient spectra at a single ultrafast time delay evolving in real lab time (purple to pink). (d-f) The ESA region highlighted in grey in (a-c) averaged together at each time point and plotted as a function of lab time along with exponential fits to the data. The fits give a photodegradation lifetime of 62 s ± 8 s.

S4. Additional spatial cavity-coupling maps and cavity transmission spectra

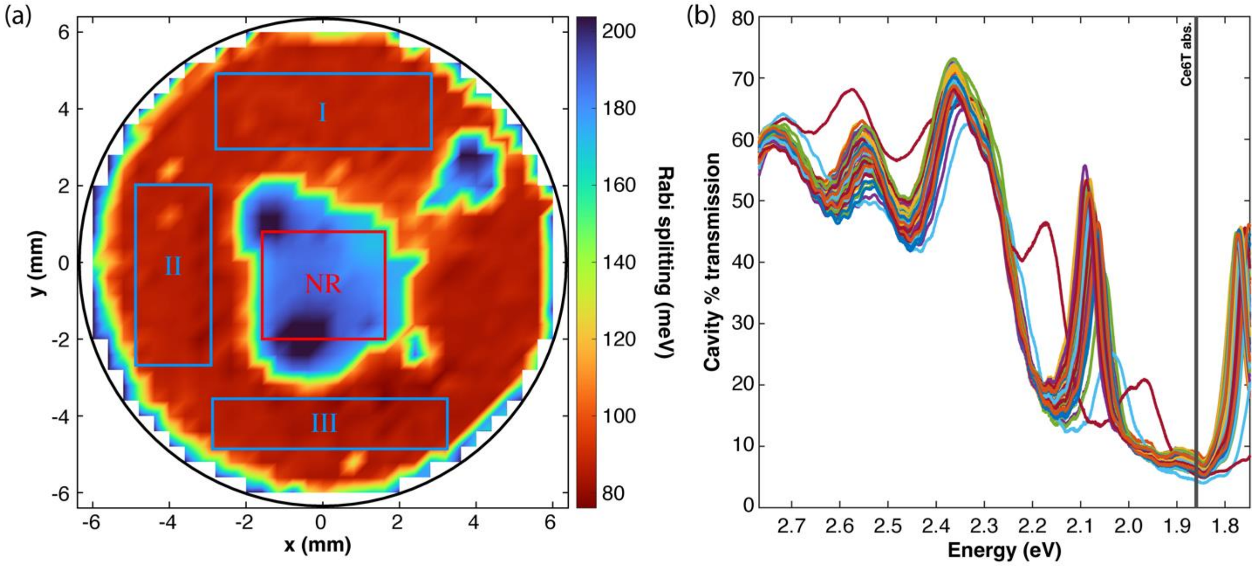

Figure S5. Alternative characterization of spatial uniformity in Cavity 1, which is filled with a 809 nm thick Ce6T/PS film, with the fourth-order longitudinal cavity mode coupled to the Ce6T Q_y band near normal incidence. (a) Spatial cavity-coupling map tracking the Rabi splitting as a function of *x* and *y* coordinates in the cavity plane. Regions of uniform cavity-coupling used for pump-probe experiments are highlighted in the blue boxes. This spatial cavity-coupling map is nearly identical to the map which tracks the LP energy in Fig. 3 of the main text, indicating that the LP is an accurate readout of the coupling conditions. Note that the center of the map, marked with a red box, represents a region where a bubble likely formed during mirror bonding. The extracted Rabi splitting in this region is not reliable. (b) Cavity transmission spectra acquired while rastering over the red region of the map labeled NR in panel (a). This area was not used for pump-probe experiments, but rather demonstrates the vastly different coupling conditions present in a single device and the importance of careful device characterization.

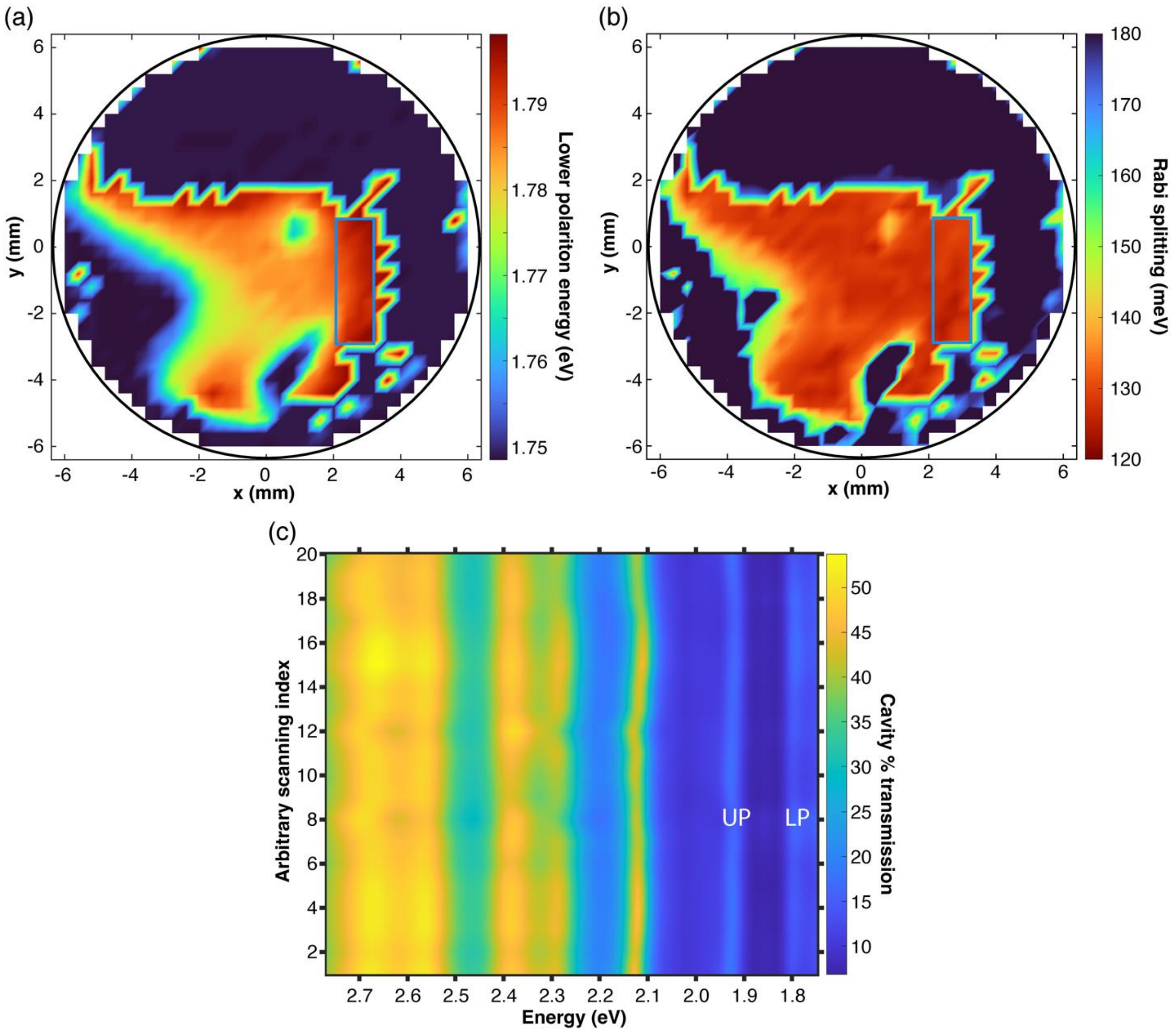

Figure S6. Characterization of spatial uniformity in Cavity 2, which is filled with a 979 nm thick Ce6T/PS film such that the fifth-order longitudinal cavity mode resonantly strong couples the Ce6T Q_y band near normal incidence. Spatial cavity-coupling maps tracking the (a) energy of the LP and (b) the Rabi splitting as a function of x and y coordinates in the cavity plane. A region of uniform cavity-coupling used for pump-probe experiments is highlighted in the blue box. (c) Cavity transmission spectra acquired while rastering over the boxed region of the map shown in panels (a) and (b). The cavity-coupling conditions do not change significantly as a function of spatial coordinates, indicating that rastering can be performed over this spatial region during a pump-probe experiment without significant cavity detuning.

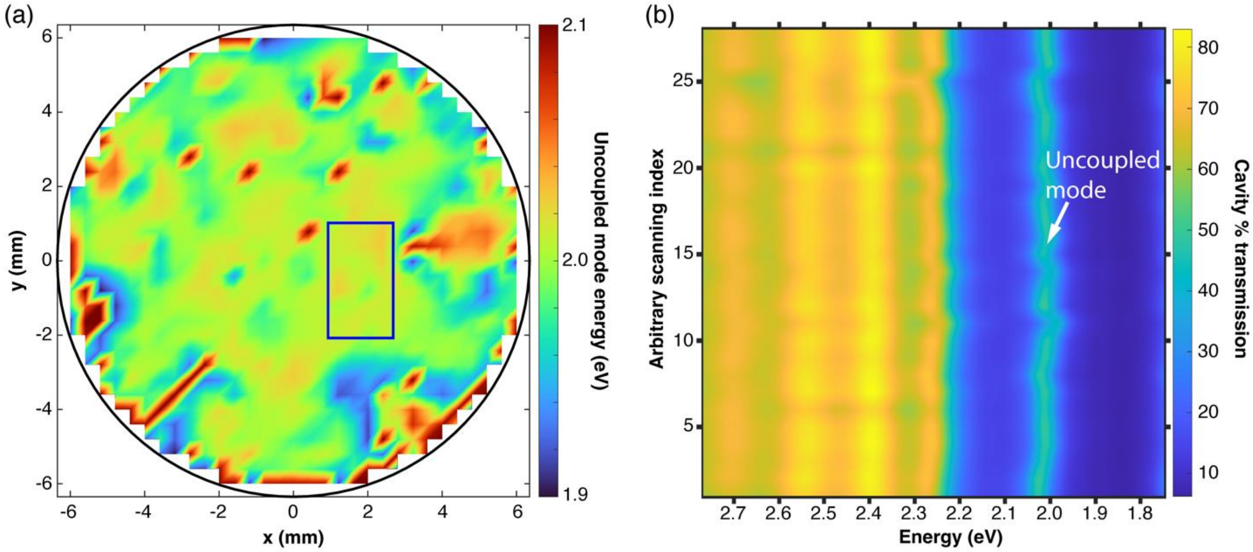

Figure S7. Spatial cavity-coupling map and pump-probe spectra of Cavity 3, which is filled with an 816 nm thick Ce6T/PS film and features a cavity mode that is red-detuned from resonance with the Ce6T Q_y band. (a) Spatial cavity-coupling map tracking the energy of the cavity mode at 2.01 eV as a function of *x* and *y* coordinates in the cavity plane. The uniform region used for rastering during pump-probe experiments is highlighted in the blue box. (b) Relatively consistent cavity transmission spectra are acquired while rastering over the boxed region of the map shown in panel (a), indicating that rastering can be performed over this region without significant cavity detuning.

S5. TCSPC and ultrafast pump-probe data for extracavity Ce6T solutions

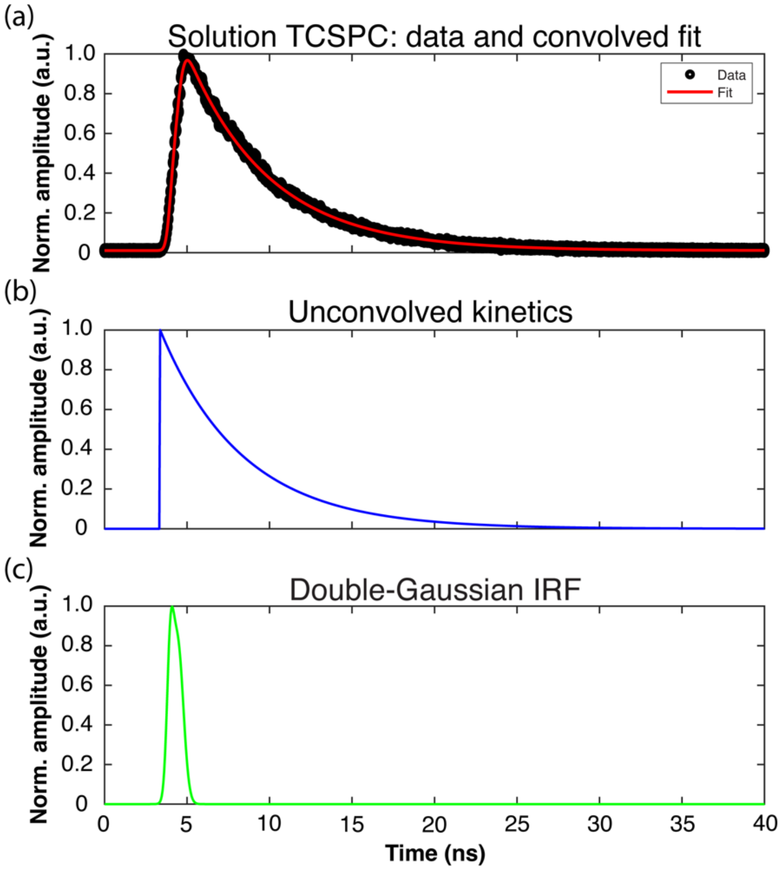

Figure S8. Ultrafast TCSPC emission data of an extracavity dilute solution of 10 µM Ce6T in toluene. Data are collected using a photoluminescence spectrometer (FLS980, Edinburgh Instruments) equipped with a picosecond pulsed diode laser (EPL-405, Edinburgh Instruments) which excites the sample at 3.09 eV (401 nm) with a pulse width of 58.1 ps. Fitting is performed using the lsqcurvefit function in MATLAB. (a) Ultrafast emission data (black dots) acquired with excitation at 3.09 eV and probing of the monomer Ce6T emission at 1.84 eV (673 nm). We fit these data (red line) to the convolution of a single exponential decay with the IRF. For these data, the IRF is obtained from the fit of the scattering signal in a solution of silica nanoparticles, as described above in Section S2. (b) The fitted exponential decay kinetics, which feature a decay time constant of 4.974 ± 0.011 ns. (c) Experimental IRF reproduced from Fig. S3.

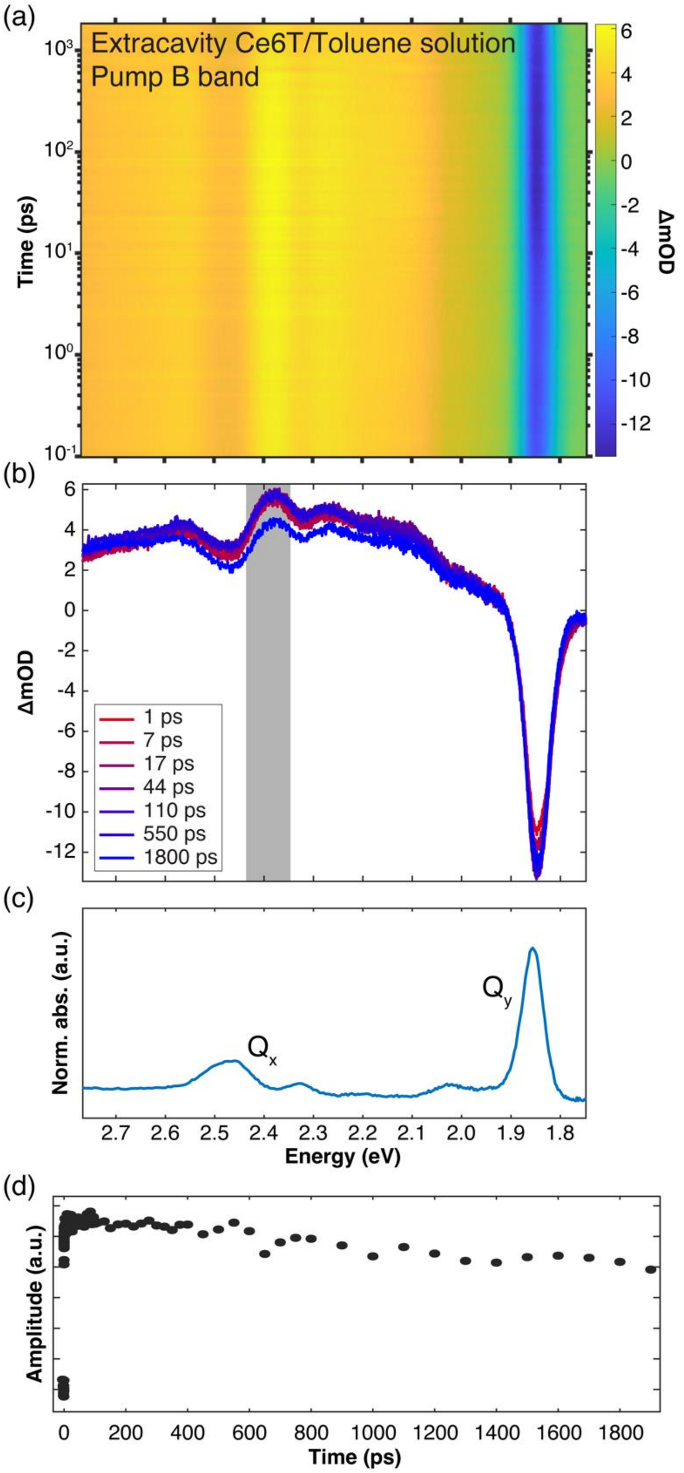

Figure S9. Transient dynamics of an extracavity solution of 100 μM Ce6T in toluene following optical excitation of the B band at 3.10 eV (400 nm). (a) Broadband pump-probe spectra and (b) representative spectral linecuts. (c) Linear absorption spectrum of the same Ce6T/toluene solution to illustrate where relevant spectral features lie. (d) Temporal linecut of the pump-probe data from panel (a) showing the ESA dynamics averaged over the spectral window from 2.35–2.42 eV (as marked in gray in panel (b)). The ESA feature is considerably longer-lived in this dilute solution than it is in dense thin films, as has been observed in prior literature, and as is also evident from the TCSPC data shown above in Fig. S8.[1–4]

S6. Ultrafast pump-probe spectra for extracavity Ce6T/PS thin films pumping the Q_y band

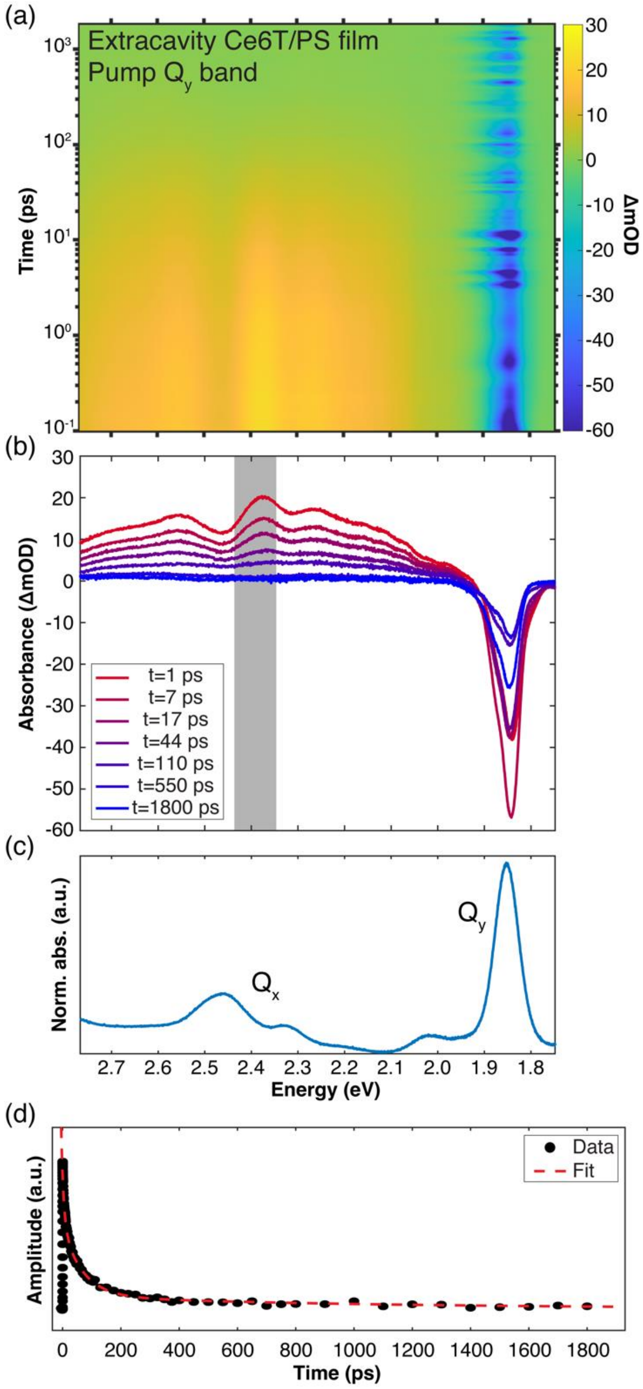

Figure S10. Transient dynamics of an extracavity Ce6T/PS film following optical excitation of the Q_y band at 1.85 eV (669 nm). (a) Broadband pump-probe spectra and (b) representative spectral linecuts. Some noise appears at 1.85 eV due to pump scatter. (c) Linear absorption spectrum of Ce6T/PS replotted from Fig. 4a to illustrate where relevant spectral features lie. (d) Temporal linecut of the pump-probe data from panel (a) showing the ESA dynamics averaged over the spectral window from 2.35–2.42 eV (as marked in gray in panel (b)). Experimental data points are shown with black dots, while the red dashed line represents a fit of these data to three parallel exponential decays.

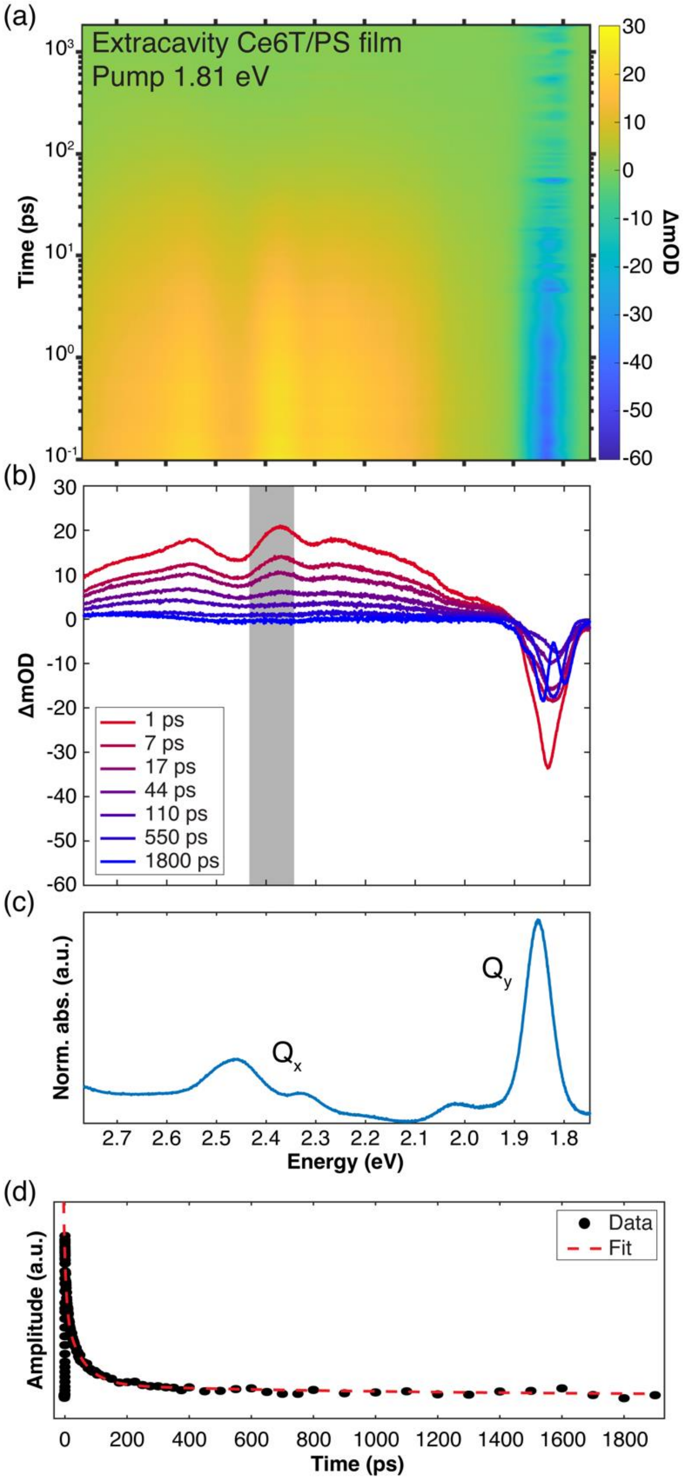

Figure S11. Transient dynamics of an extracavity Ce6T/PS film following optical excitation red-detuned from the Q_y band at 1.81 eV (684 nm). This represents an extracavity control experiment for excitation of the lower polariton in intracavity experiments. (a) Broadband pump-probe spectra and (b) representative spectral linecuts. Some noise appears at 1.81 eV due to pump scatter. (c) Linear absorption spectrum of Ce6T/PS replotted from Fig. 4a to illustrate where relevant spectral features lie. (d) Temporal linecut of the pump-probe data from panel (a) showing the ESA dynamics averaged over the spectral window from 2.35–2.42 eV (as marked in gray in panel (b)). Experimental data points are shown with black dots, while the red dashed line represents a fit of these data to three parallel exponential decays.

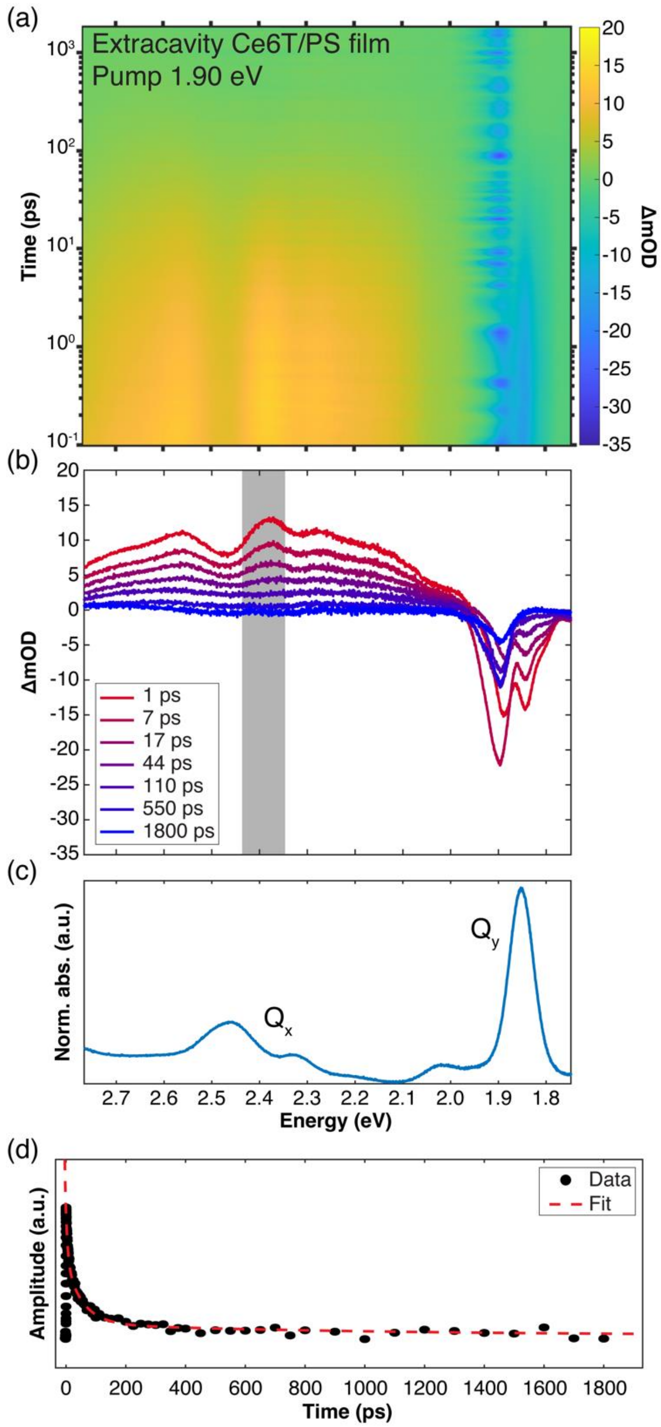

Figure S12. Transient dynamics of an extracavity Ce6T/PS film following optical excitation blue-detuned from the Q_y band at 1.90 eV (653 nm). This represents an extracavity control experiment for excitation of the upper polariton in intracavity experiments. (a) Broadband pump-probe spectra and (b) representative spectral linecuts. Some noise appears at 1.90 eV due to pump scatter. (c) Linear absorption spectrum of Ce6T/PS replotted from Fig. 4a to illustrate where relevant spectral features lie. (d) Temporal linecut of the pump-probe data from panel (a) showing the ESA dynamics averaged over the spectral window from 2.35–2.42 eV (as marked in gray in panel (b)). Experimental data points are shown with black dots, while the red dashed line represents a fit of these data to three parallel exponential decays.

S7. Excimer TCSPC data and ultrafast pump-probe spectra of excimer stimulated emission (SE) for extracavity Ce6T/PS thin films

We acquire TCSPC traces for six extracavity Ce6T/PS films, exciting the sample at 3.09 eV (401 nm) and probing at 1.68 eV (740 nm) to characterize excimer emission dynamics. Data from one representative film is shown in Fig. S13 below, wherein we fit the TCSPC data to the convolution of the IRF and two parallel decaying exponentials. The thin film TCSPC data kinetics are much faster than those of solution-phase Ce6T. As a result, we find that representing the IRF exactly via spline fits of the experimental silica nanoparticle scattering data (e.g. black dots in Fig. 3) yields better fits than representing the IRF with two fitted Gaussians (e.g. red trace in Fig. 3). We find decay time constants of 230 ± 30 ps and 1170 ± 120 ps for the excimer TCSPC signal in extracavity Ce6T/PS films. Our analysis of the excimer kinetics differs from that of Biswas et al.[5] who instead report a rise of 200 ps followed by a decay of 1.1 ns. When we fit our TCSPC data to a convolution of the experimental IRF with an exponential rise and two decays, we find a much shorter rise time constant of ~20 ps ± 20 ps which TCSPC does not have sufficient time resolution to capture. As a result, we do not include a rise in our TCSPC fits.

We find consistent results using ultrafast pump-probe spectroscopy to track stimulated emission (SE) signatures that report on the same excimer dynamics (see Fig. S14 below and Section III.B of the main text). We analyze the pump-probe SE data using similar practices as for analysis of the ESA dynamics. We take a temporal linecut of these data averaged over the spectral window from 1.67–1.72 eV. We trim this linecut before 1.5 ps and fit the longer-time dynamics to an exponential rise and two parallel decays (plus a constant offset) using the nonlinear least squares method in MATLAB. Results from individual fits to our data can be found in the accompanying Excel sheet. Fitting the SE data to an exponential rise and two parallel decays, we find an excimer rise time of 7.9 ± 0.5 ps, and decay time constants of 240 ± 30 ps and 1300 ± 110 ps, completely consistent with the TCSPC data. Our use of two independent spectroscopic techniques lends confidence that we are correctly capturing the Ce6T excimer rise and decay dynamics. In addition, our quoted ~8 ps excimer rise time is more consistent with the excimer formation time observed in other non-diffusion-limited systems.[6–9]

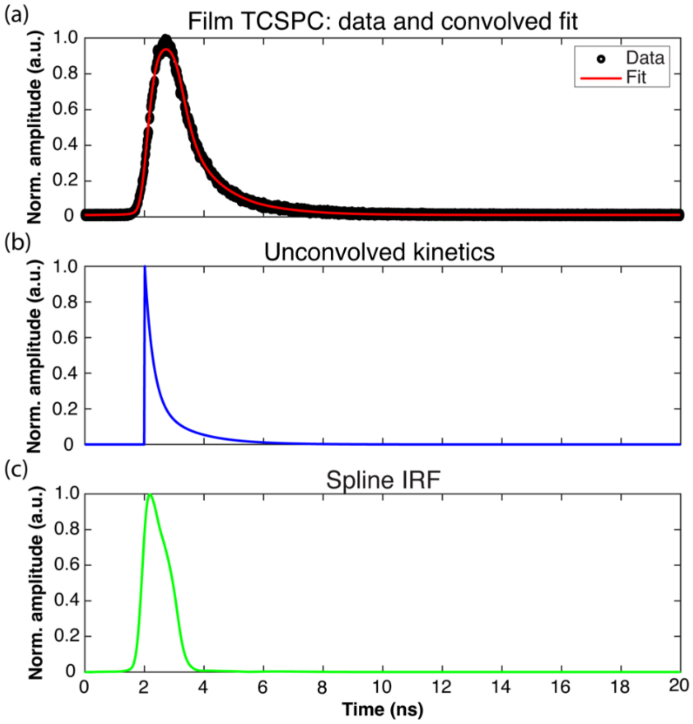

Figure S13. Ultrafast TCSPC emission data for a Ce6T/PS thin film. Data are collected using a photoluminescence spectrometer (FLS980, Edinburgh Instruments) fitted with a picosecond pulsed diode laser (EPL-405, Edinburgh Instruments) which excites the sample at 3.09 eV (401 nm) with a pulse width of 58.1 ps. Fitting is performed using the lsqcurvefit function in MATLAB. (a) Ultrafast emission data (black dots) acquired with 3.09 eV excitation and probing of the Ce6T excimer emission at 1.68 eV (740 nm). We fit (red line) these data to the convolution of two parallel exponential decays with the experimental IRF. For these data, the experimental IRF was represented by fitting a spline to the exact experimental IRF lineshape obtained from the scattering signal of a silica nanoparticle solution. This was done to capture smaller transient features in the scattering signal that bias the shorter emission decay in thin films. (b) The extracted Ce6T TCSPC kinetics after fitting. (c) The experimental IRF acquired by averaging together three spline fits of silica nanoparticle scattering data.

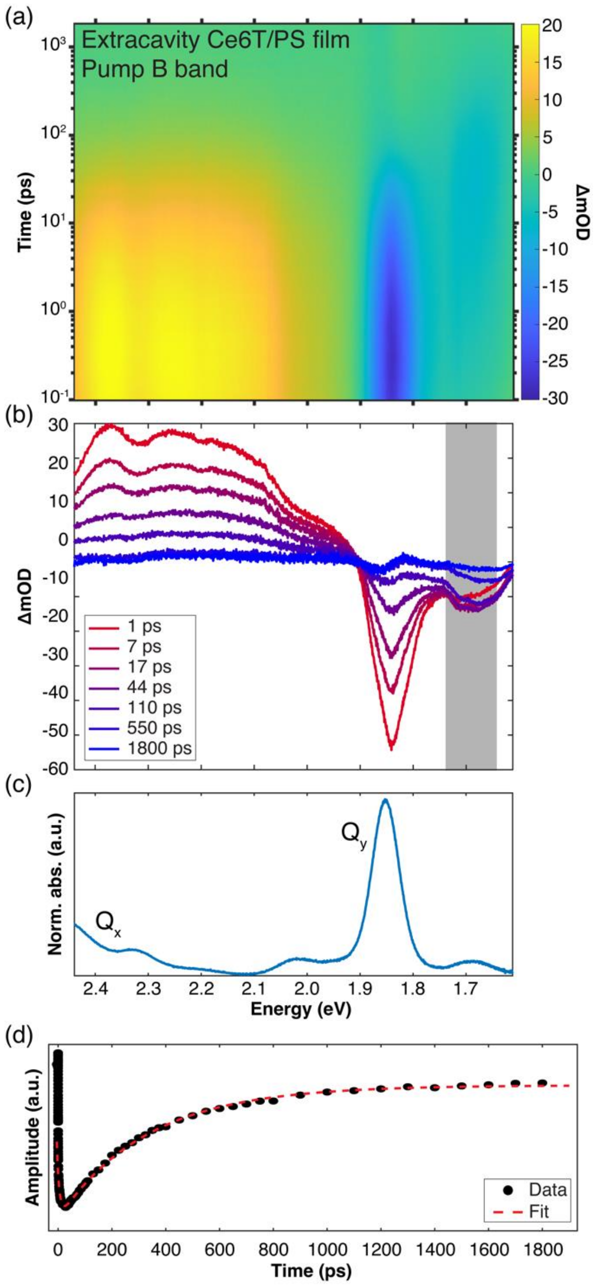

Figure S14. Transient dynamics of an extracavity Ce6T/PS film following optical excitation of the B band at 3.10 eV (400 nm), with an emphasis on the signatures of excimer stimulated emission (SE) near 1.7 eV. (a) Broadband pump-probe spectra and (b) representative spectral linecuts. (c) Linear absorption spectrum of Ce6T/PS replotted from Fig. 4a to illustrate where relevant spectral features lie. (d) Temporal linecut of the pump-probe data from panel (a) showing the SE dynamics averaged over the spectral window from 1.67– 1.72 eV (as marked in gray in panel (b)). Experimental data points are shown with black dots, while the red dashed line represents a fit of these data to an exponential rise and two parallel exponential decays.

S8. Kinetic model for excited-state dynamics in Ce6T/PS thin films

We derive a kinetic model for the excited-state dynamics of Ce6T in PS thin films following the work of Kushida *et al.*[1] In the following, M denotes ground-state Ce6T monomers in the S_0 electronic ground state, M^* denotes excited-state Ce6T monomers in the Q_y manifold, and MM^* denotes an excited-state excimer complex:

$$M + h\upsilon \rightarrow M^* \tag{S1}$$

$$M^* \xrightarrow{k_r + k_{nr}} M\ (+h\upsilon) \tag{S2}$$

$$M + M^* \xrightarrow{k_{ex}} MM^* \tag{S3}$$

$$MM^* \xrightarrow{k_{ex\ decay}} 2M\ (+h\upsilon) \tag{S4}$$

The above set of equations assumes that [*M*] is large and constant and that back-reactions are negligible at each step. Further, it does not consider monomer-excimer interactions. Within this framework, the rate equation for the excited monomer population is:

$$\frac{d[M^*]}{dt} = -(k_r + k_{nr} + k_{ex}[M])\ [M^*] \tag{S5}$$

while the excimer population evolves according to:

$$\frac{d[MM^*]}{dt} = k_{ex}[M][M^*] - k_{ex\ decay}[MM^*] \tag{S6}$$

We determine the monomer relaxation rate $k_r + k_{nr}$ from TCSPC measurements of dilute Ce6T solutions in toluene. We assume this quantity to be preserved in thin films, consistent with the nearly identical monomer decay lifetimes reported by Kushida *et al.* and Biswas *et al.* in solution and in thin films, respectively.[1,5]

To obtain the excimer formation rate k_{ex}, we use Eq. S6 to derive the time-dependent excimer concentration shown below, where A is an amplitude prefactor:

$$[MM^*(t)] = A \cdot \left(e^{-(k_r + k_{nr} + k_{ex}[M])t} - e^{-k_{ex\ decay}t}\right) \tag{S7}$$

The stimulated emission we observe in extracavity ultrafast pump-probe experiments is a direct readout of the time-dependent excimer concentration, so we can fit these data to find k_{ex}. As excimer formation is much faster than either monomer decay or excimer decay, we take [MM^*] at early ultrafast time delays to report only on excimer formation kinetics. This allows us to reduce Eq. S7 to a single exponential which can then be fit to the stimulated emission data, giving:

$$k_{ex} = \frac{\frac{1}{\tau_{rise}} - (k_r + k_{nr})}{[M]} \tag{S8}$$

where $\boldsymbol{\tau_{rise}}$ is the stimulated emission formation time constant of excimer formation, $\boldsymbol{k_r} + \boldsymbol{k_{nr}}$ is known from dilute TCSPC measurements, and $[\boldsymbol{M}]$ is known for our thin film samples.

To calculate excimer relaxation, we take $\boldsymbol{k_{ex\ decay\ 1}}$ and $\boldsymbol{k_{ex\ decay\ 2}}$ as two separate rate constants required to fit the longer-time excimer decays in the TCSPC and stimulated emission data. We obtain each rate constant by simply taking the inverse of fitted time constants. This is justified in that: (1) excimer formation is much faster than excimer decay allowing us to approximate the late time points to be independent of excimer formation; and (2) two parallel decays of the excimers are consistent with the inhomogeneity of known local environments discussed in Section 3.2.1 of the main text.

We report the resulting $\boldsymbol{k_r} + \boldsymbol{k_{nr}}$, $\boldsymbol{k_{ex\ decay\ 1}}$, and $\boldsymbol{k_{ex\ decay\ 2}}$ rate constants below in Figure S15. Our rate constants are similar to those reported by Kushida *et al.*, validating the approximations we make here to calculate these values. Notably, our excimer formation rate constant, $\boldsymbol{k_{ex}}$, value is much greater than the value Kushida *et al.* observe for solution-phase Ce6T. This is expected due to the pre-organization of Ce6T molecules in thin films and lack of a diffusion-limited kinetics in thin films, as we discuss further in Section 3.2.2. of the main text.

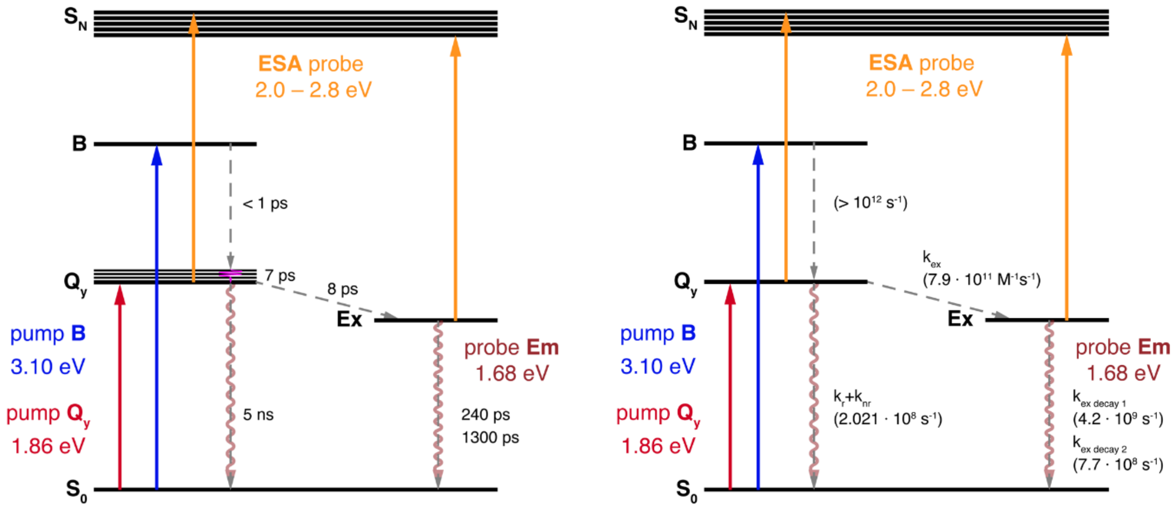

Figure S15. Energy diagrams labeled with observed time constants (left) and extracted rate constants (right) for Ce6T in PS thin films. Reported time constants are acquired from excited-state absorption (ESA) and emission (Em) signatures from pump-probe and TCSPC experiments. Rate constants are obtained using the model described above. Solid straight lines indicate optical excitation, solid wavy lines indicate excited-state vibrational cooling or fluorescence readout, and dashed lines indicate nonradiative relaxation.

S9. Ultrafast pump-probe spectra of Cavities 2 and 3 pumping the B band at 3.10 eV

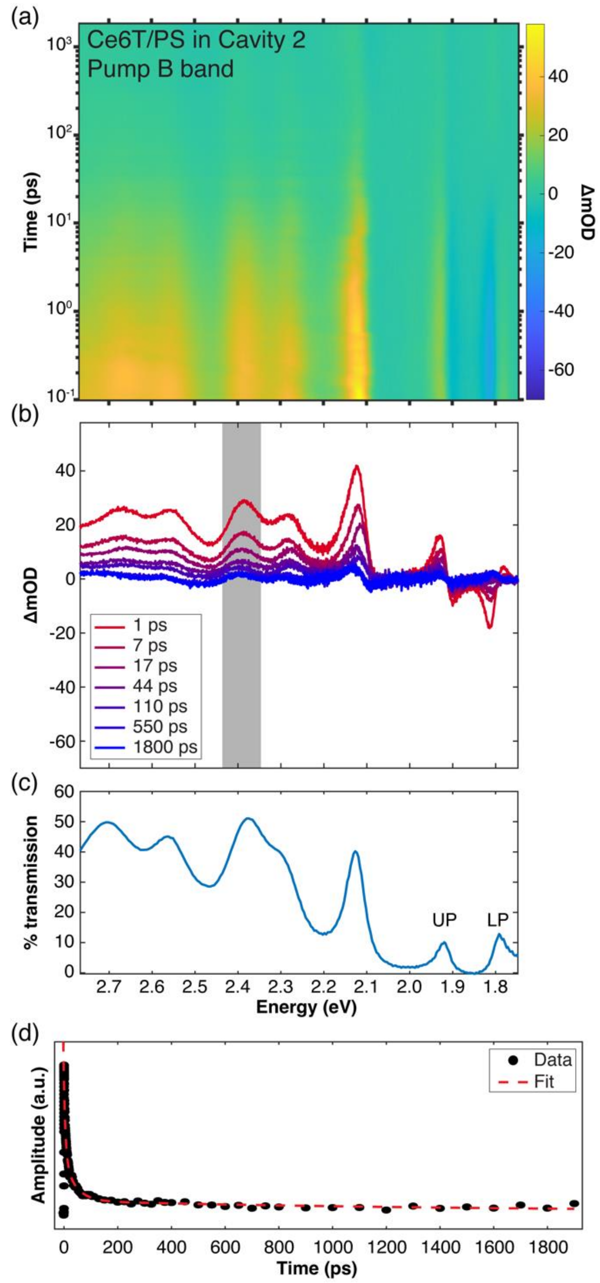

Figure S16. Transient dynamics of a Ce6T/PS film under strong coupling of the Q_y band in DBR Cavity 2 following optical excitation of the B band at 3.10 eV (400 nm). These data are nearly identical to those acquired for Cavity 1 shown in Fig. 8 of the main text. (a) Broadband pump-probe spectra and (b) representative spectral linecuts. Excited-state absorption (ESA) features are clearly visible from 2.2–2.8 eV through the transparent region of the DBR mirrors. Two derivative-like features appear at the energies of the polaritons on either side of the Q_y band. These arise due to a contraction of the Rabi splitting from bleaching of the $S_0 \rightarrow Q_y$ transition. An additional derivative-like lineshape at 2.1 eV is representative of an uncoupled cavity mode shifting in energy due to modulation of the intracavity background refractive index. The Q_y bleach feature at 1.86 eV is missing in these data compared to the pump-probe spectra in Cavity 1 where it is readily apparent. This is likely due to the larger Rabi splitting of Cavity 2 which places the polariton transmission windows of the cavity further from the Q_y bleach, filtering any signature of the bleach out with greater coupling strength. (c) Linear transmission spectrum of Cavity 2 replotted from Fig. 7c of the main text. (d) Temporal linecut of the pump-probe data from panel (a) showing the ESA decay averaged over the spectral window from 2.35–2.42 eV (as marked in gray in panel (b)). Experimental data points are shown with black dots, while the red dashed line represents a fit of these data to three parallel exponential decays.

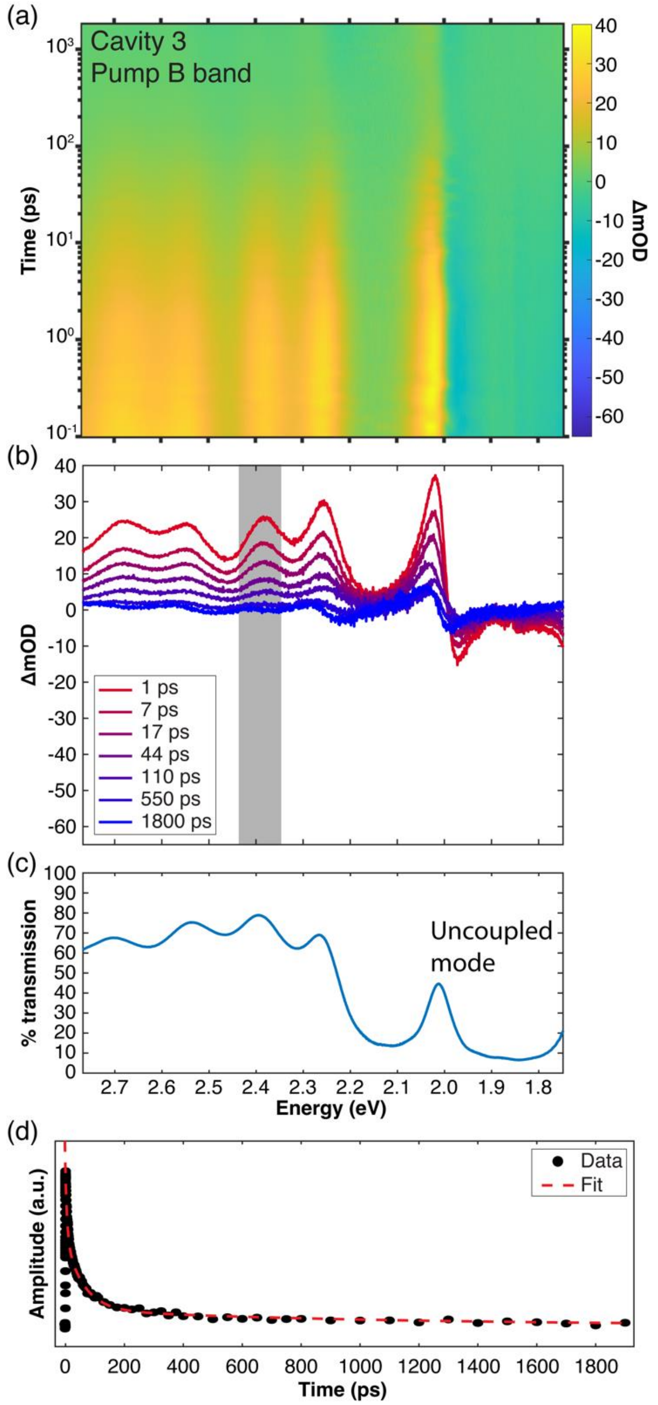

Figure S17. Transient dynamics of a Ce6T/PS film in Cavity 3 following optical excitation of the B band at 3.10 eV (400 nm). (a) Broadband pump-probe spectra and (b) representative spectral linecuts. Excited-state absorption (ESA) features are clearly visible from 2.2−2.8 eV through the transparent region of the DBRs. (c) Linear transmission spectrum of Cavity 3, showing the off-resonance cavity mode near 2.01 eV. (d) Temporal linecut of the pump-probe data from panel (a) showing the ESA decay averaged over the spectral window from 2.35–2.42 eV (as marked in gray in panel (b)). Experimental data points are shown with black dots, while the red dashed line represents a fit of these data to three parallel exponential decays.

S10. Ultrafast pump-probe spectra of Cavity 1 pumping the lower and upper polaritons at 1.81 eV and 1.90 eV

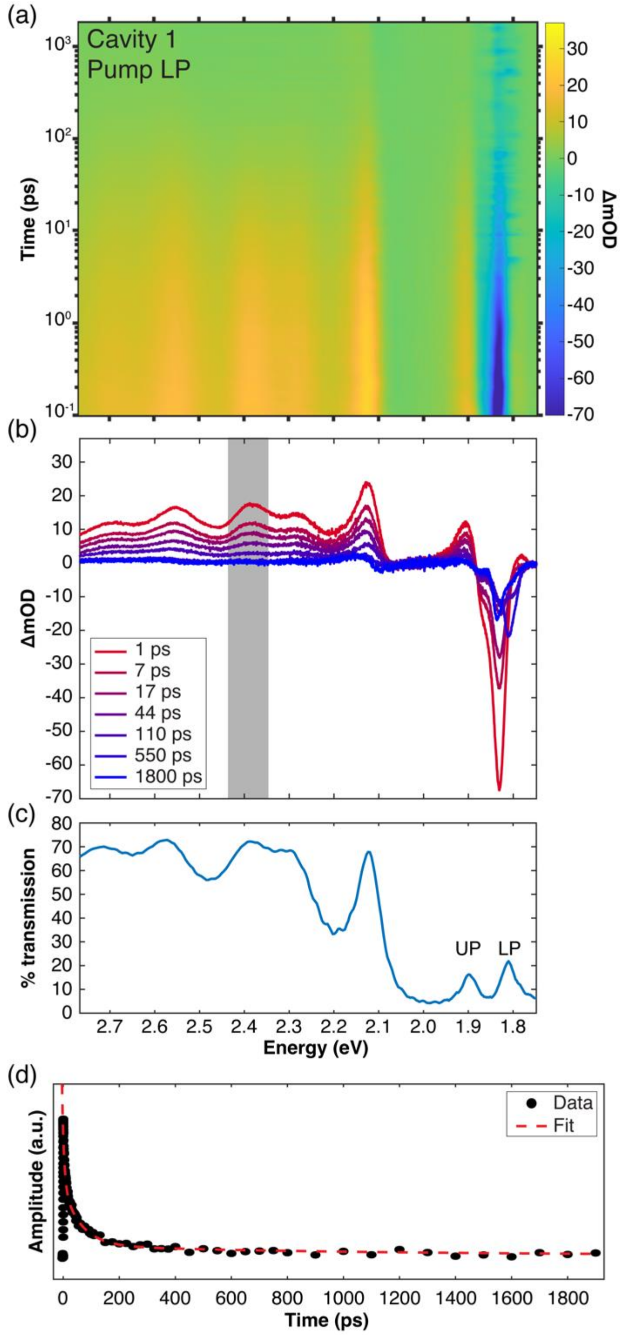

Figure S18. Transient dynamics of a Ce6T/PS film under strong coupling of the Q_y band in DBR Cavity 1 following optical excitation of the lower polariton (LP) at 1.81 eV (684 nm). (a) Broadband pump-probe spectra and (b) representative spectral linecuts. Excited-state absorption (ESA) features are visible from 2.2−2.8 eV through the transparent region of the DBR mirrors. (c) Linear transmission spectrum of Cavity 1 replotted from Fig. 7a of the main text. (d) Temporal linecut of the pump-probe data from panel (a) showing the ESA decay averaged over the spectral window from 2.35–2.42 eV (as marked in gray in panel (b)). Experimental data points are shown with black dots, while the red dashed line represents a fit of these data to three parallel exponential decays.

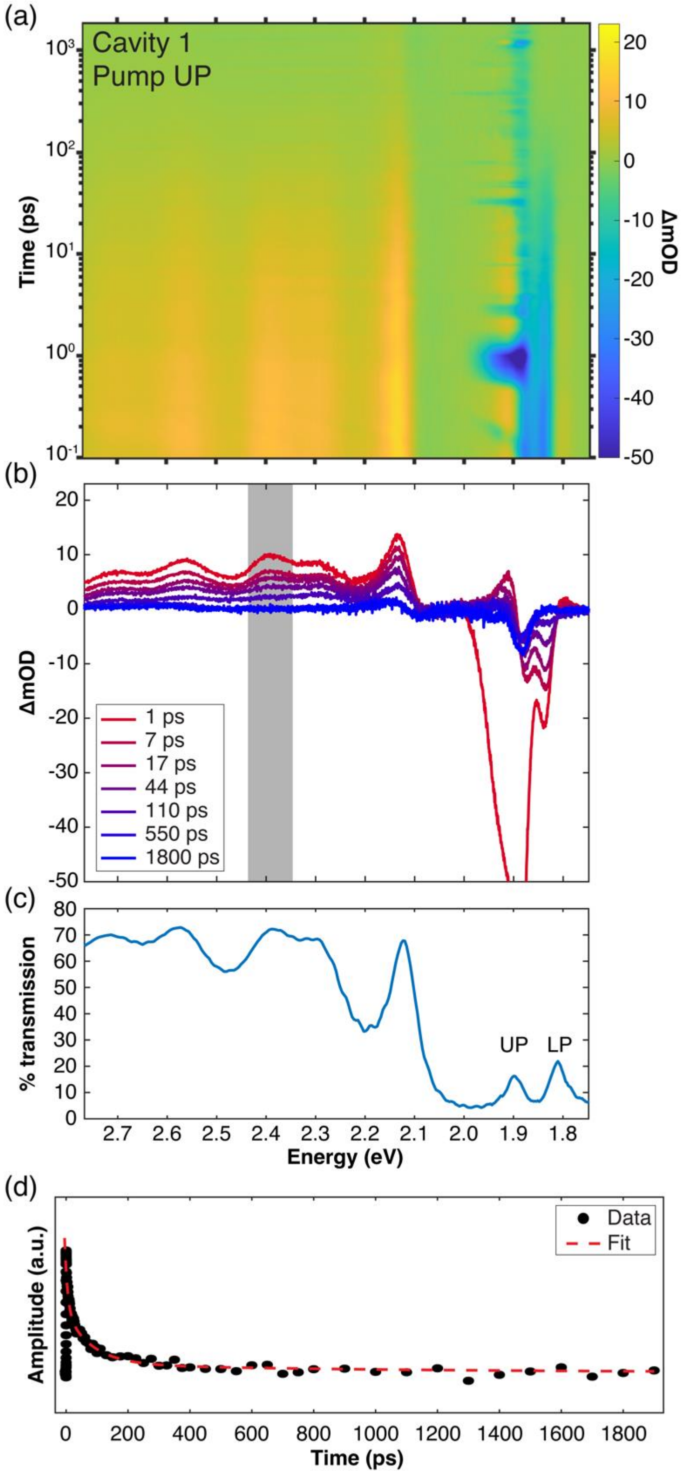

Figure S19. Transient dynamics of a Ce6T/PS film under strong coupling of the Q_y band in DBR Cavity 1 following optical excitation of the upper polariton (UP) at 1.90 eV. (a) Broadband pump-probe spectra and (b) representative spectral linecuts. Excited-state absorption (ESA) features are visible from 2.2−2.8 eV through the transparent region of the DBR mirrors. (c) Linear transmission spectrum of Cavity 1 replotted from Fig. 7a of the main text. (d) Temporal linecut of the pump-probe data from panel (a) showing the ESA decay averaged over the spectral window from 2.35–2.42 eV (as marked in gray in panel (b)). Experimental data points are shown with black dots, while the red dashed line represents a fit of these data to three parallel exponential decays.

S11. Ultrafast response of the UV fused silica substrate, a single DBR mirror, a PS thin film, and an empty cavity

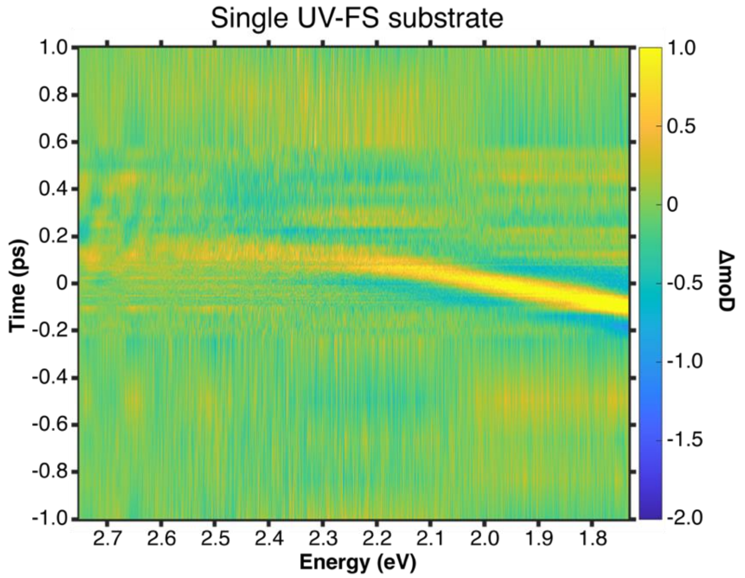

Figure S20. Transient pump-probe spectrum of a single 3 mm thick UV-fused silica (UV-FS) substrate acquired with a 3.10 eV pump. These data show no significant transient signals except for a pump-probe overlap feature near time zero. These data are not chirp corrected.

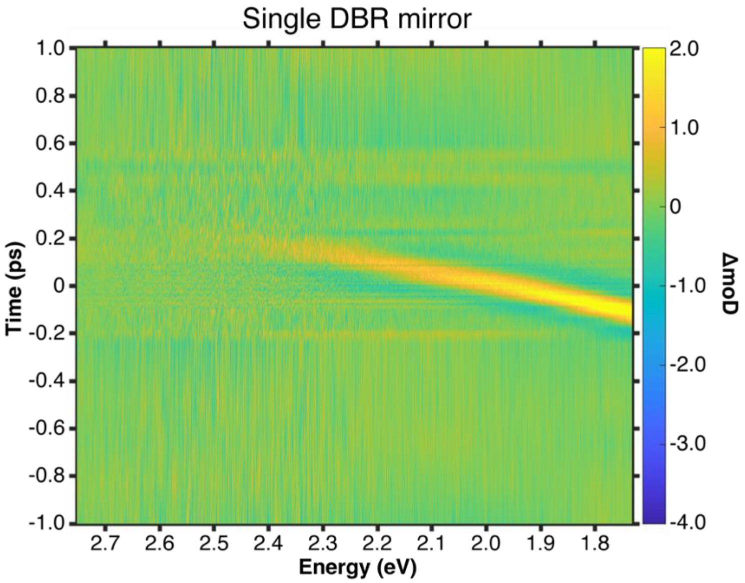

Figure S21. Transient pump-probe spectrum of a single DBR mirror acquired with a 3.10 eV pump pulse. These data show no significant transient signals except for a pump-probe overlap feature near time zero. These data are not chirp corrected.

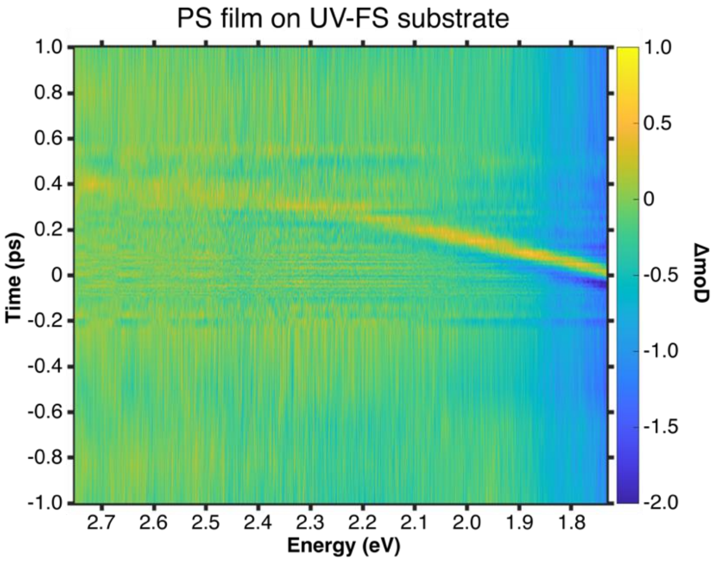

Figure S22. Transient pump-probe spectrum of a bare 705 nm polystyrene (PS) film on UV-FS acquired with a 3.10 eV pump. These data show no significant transient signals except for a pump-probe overlap feature near time zero. These data are not chirp corrected.

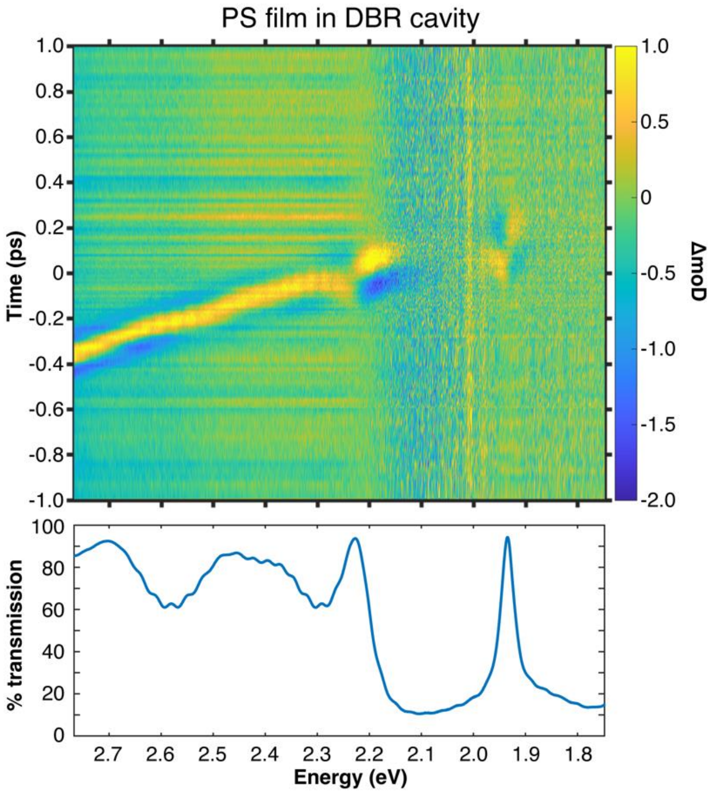

Figure S23. Transient pump-probe spectrum of a two-mirror DBR cavity containing just a 799 nm polystyrene (PS) film, acquired with a 3.10 eV pump. These data show no significant signals except for a short overlap feature near time zero. These data are not chirp corrected. The noise evident at probe energies below 1.9 eV and from 2.0−2.2 eV is due to low transmission of light through the cavity at these energies. The lower panel plots the linear cavity transmission spectrum to illustrate the position of a cavity fringe in the DBR mirror stop band at ~1.93 eV.

REFERENCES

(1) Kushida, S.; Wang, K.; Seidel, M.; Yamamoto, Y.; Genet, C.; Ebbesen, T. W. Fluidic Molecular Dynamics and Energy Relaxation Pathways in Solution-State Electronic Strong Coupling Using a High-Mode-Number Cavity. J. Phys. Chem. Lett. 2025, 16 (33), 8570. https://doi.org/10.1021/acs.jpclett.5c02008.

(2) Kolman, A.; Pedzinski, T.; Lewandowska-Andralojc, A. Spectroscopic Insights into BSA-Mediated Deaggregation of m-THPC. Sci. Rep. 2024, 14 (1), 22343. https://doi.org/10.1038/s41598-024-73266-2.

(3) Yang, Y.; Zhang, L.; Xiao, C.; Huang, Z.; Zhao, F.; Yin, J. Highly Efficient Upconversion Photodynamic Performance of Rare-Earth-Coupled Dual-Photosensitizers: Ultrafast Experiments and Excited-State Calculations. Nanophotonics 2024, 13 (4), 443. https://doi.org/10.1515/nanoph-2023-0772.

(4) Duncan, K. M.; Watt, D. R.; Knowlton, W. B.; Turner, D. B.; Mass, O. A.; Pensack, R. D. Singlet Excited-State Lifetimes of Nitro- and Dimethylamino-Substituted Bacteriochlorins Vary in Their Sensitivity to Solvent Polarity. J. Phys. Chem. C 2025, 129, 11439. https://doi.org/10.1021/acs.jpcc.5c01649.

(5) Biswas, S.; Mondal, M.; Chandrasekharan, G.; Mony, K. S.; Singh, A.; Thomas, A. Electronic Strong Coupling Modifies the Ground-State Intermolecular Interactions in Self-Assembled Chlorin Molecules. Nat. Commun. 2025, 16 (1), 5115. https://doi.org/10.1038/s41467-025-60025-8.

(6) Pensack, R. D.; Ashmore, R. J.; Paoletta, A. L.; Scholes, G. D. The Nature of Excimer Formation in Crystalline Pyrene Nanoparticles. J. Phys. Chem. C 2018, 122 (36), 21004. https://doi.org/10.1021/acs.jpcc.8b03963.

(7) Brown, K. E.; Salamant, W. A.; Shoer, L. E.; Young, R. M.; Wasielewski, M. R. Direct Observation of Ultrafast Excimer Formation in Covalent Perylenediimide Dimers Using Near-Infrared Transient Absorption Spectroscopy. J. Phys. Chem. Lett. 2014, 5 (15), 2588. https://doi.org/10.1021/jz5011797.

(8) Margulies, E. A.; Shoer, L. E.; Eaton, S. W.; Wasielewski, M. R. Excimer Formation in Cofacial and Slip-Stacked Perylene-3,4:9,10-Bis(Dicarboximide) Dimers on a Redox-Inactive Triptycene Scaffold. Phys. Chem. Chem. Phys. 2014, 16 (43), 23735. https://doi.org/10.1039/C4CP03107E.

(9) Giaimo, J. M.; Lockard, J. V.; Sinks, L. E.; Scott, A. M.; Wilson, T. M.; Wasielewski, M. R. Excited Singlet States of Covalently Bound, Cofacial Dimers and Trimers of Perylene-3,4:9,10-Bis(Dicarboximide)s. J. Phys. Chem. A 2008, 112 (11), 2322. https://doi.org/10.1021/jp710847q.
